# Effect of High-Flow Nasal Cannula on Sleep-disordered Breathing and Sleep Quality in Patients With Acute Stroke

**DOI:** 10.7759/cureus.9303

**Published:** 2020-07-20

**Authors:** Nobuto Nakanishi, Yasuhiro Suzuki, Manabu Ishihara, Yoshitoyo Ueno, Natsuki Tane, Yumiko Tsunano, Taiga Itagaki, Jun Oto

**Affiliations:** 1 Emergency and Critical Care Medicine, Tokushima University Hospital, Tokushima, JPN; 2 Emergency and Critical Care Center, Kurashiki Central Hospital, Kurashiki, JPN; 3 Emergency and Disaster Medicine, Tokushima University Hospital, Tokushima, JPN

**Keywords:** high-flow nasal cannula, sleep-disordered breathing, stroke, adherence, oxygen desaturation index, sleep efficiency, rapid eye movement sleep, apnea-hypoxia index

## Abstract

Introduction

Sleep-disordered breathing (SDB) is common after stroke. Although the standard treatment of SDB is continuous positive airway pressure (CPAP) ventilation, the patient’s intolerance and discomfort result in low adherence rates. Alternatively, high-flow nasal cannula (HFNC) may be useful as it reduces upper airway collapse with low level of positive pressure and well tolerability. The aim of this study was to investigate whether HFNC therapy reduces SDB and improves sleep quality with higher compliance rate.

Methods

We included acute stroke patients with SDB for the assessment of apnea-hypopnea index (AHI) >5/h using WatchPAT 200 (Itamar Medical Ltd, Caesarea, Israel). Patients who met inclusion criteria received HFNC therapy (40 L/min) with monitoring by WatchPAT. AHI, oxygen desaturation index (ODI), sleep efficiency, and rapid eye movement (REM) sleep were compared in patients with and without HFNC therapy. We also evaluated the patient’s comfort of HFNC therapy (discomfort or not).

Results

Among 17 patients assessed for AHI, 12 received HFNC therapy. HFNC therapy was not adhered in two patients due to intolerance. Eight patients remained for final analysis. There were no differences in SDB and sleep quality with and without HFNC therapy as follows: HFNC therapy vs control; AHI 24.9 ± 20.1 vs 21.3 ± 15.0/h (p = 0.63), ODI 16.2 ± 16.5 vs 12.9 ± 12.3/h (p = 0.54), sleep efficiency 80.4 ± 12.9 vs 87.1 ± 6.2 (p = 0.28), percentage of REM sleep 19.4% ± 9.6% vs 27.6% ± 8.9% (p = 0.07). Two patients (17%) complained of discomfort among eight patients.

Conclusion

HFNC therapy did not improve SDB and sleep quality. Nonadherence and discomfort were observed in HFNC therapy. We need a large trial to confirm this result.

## Introduction

Sleep-disordered breathing (SDB) is common after stroke, which is primarily characterized by upper airway obstruction (obstructive sleep apnea syndrome: OSA), and only 7% of patients primarily having loss of central respiratory drive (central sleep apnea syndrome: central SAS) [1] .The prevalence of SDB after stroke is up to 72% compared to 33% in the general population [1,2]. SDB occurring after stroke has been shown to increase stroke-related mortality and morbidity [3]. The presence of SDB in stroke patients is associated with worse functional outcomes, longer duration of hospitalization, and more psychiatric and cognitive dysfunction [4].

The current standard of treatment for SDB is continuous positive airway pressure (CPAP) ventilation [5]. Emerging evidence suggests that CPAP ventilation therapy in poststroke patients leads to faster functional recovery and reduction in length of hospital stay and frequency of rehospitalization [6,7]. Moreover, it has been reasonably assumed that early respiratory support is more effective than late intervention in acute stroke patients. However, 25%-50% of patients with SDB will refuse or not tolerate the use of CPAP ventilation therapy due to complications and discomfort [8]. Alternatively, high-flow nasal cannula (HFNC) therapy may be useful as it can reduce upper airway collapse with low level of positive pressure [9]. Moreover, HFNC therapy washes out nasopharyngeal dead space and decreases the level of carbon dioxide [10]. Because of low pressure and no requirement of tight-fitting mask, HFNC therapy may achieve a higher compliance rate in SDB treatment.

We hypothesized that HFNC therapy can attenuate SDB and improve SDB and sleep quality in acute stroke patients with higher compliance rate. Moreover, no previous study has investigated the effect of HFNC therapy on SDB in acute stroke patients despite the associated morbidity and mortality [3]. In the present study, we investigated the effect of HFNC therapy on the severity of SDB and sleep characteristics in patients with acute stroke.

## Materials and methods

Study design

From May 2017 to February 2018, we conducted a pilot study at the Stroke Care Unit of Tokushima University Hospital. This study was approved by the clinical research ethics committee of Tokushima University Hospital (approval number: 2665) and registered as a clinical trial (UMIN-Clinical Trials Registry: 000040190). Written informed consent was obtained from patients or their authorized surrogate decision-makers.

Patient population

During the study period, we screened patients with suspected SDB using nursing records at the Stroke Care Unit in Tokushima University Hospital. If patients were considered to have SDB, the screening test was conducted at nighttime for 8 h to evaluate the apnea-hypopnea index (AHI) using a wearable sleep monitor. Patients who met the criteria of AHI >5/h were included in this study. We excluded patients based on the following exclusion criteria: age <18 years, SAS with suspected other conditions (primary neuromuscular disease, heart failure, and narcolepsy), past history of SAS, patients having unsuccessful recording in any WatchPAT channel, and the influence to WatchPAT, including catecholamine use, permanent pacemaker, and sustained nonsinus arrhythmia.

High-flow nasal cannula

On the next day of screening, HFNC therapy was performed at nighttime for 8 h, which is the same duration used for the screening measurement. In HFNC therapy, we used the Optiflow^TM^ system, which incorporated an O_2_/air blender and a heated humidifier (MR850, Fisher & Paykel Healthcare, Auckland, New Zealand). The MR850 was set in an invasive mode at a temperature of 40°C. The flow was set at 40 L/min, and then the flow was titrated to 30 L/min as the minimum flow to generate a pneumatic sprint effect according to the patient’s acceptance. The fraction of inspired oxygen was set at the beginning to maintain an SpO_2_ level of >93%.

WatchPAT

To assess the severity of SDB and sleep quality, we used the WatchPAT (WatchPAT 200U, Itamar Medical Ltd, Caesarea, Israel) on two consecutive nights at the first screening and during HFNC therapy. The WatchPAT is a device worn around the wrist with a finger-mounted probe and a snoring sensor on the chest. The device has been approved by the Food and Drug Administration and used as a type 3 monitoring device by the American Academy of Sleep Medicine (AASM) [11]. An automatic algorithm analyzes the WatchPAT data using its original software (ZZZ PAT version 4.4.65.3, Itamar Medical Ltd, Caesarea, Israel). The WatchPAT can measure peripheral arterial tone (PAT), heart rate, oxygen saturation, body position, respiratory disturbance index (RDI), AHI, 4% oxygen desaturation index (ODI), total sleep time, sleep efficiency, rapid eye movement (REM) sleep, and non-REM sleep. RDI is calculated from the number of respiratory events per hour, which meets any of the following three criteria: (1) reduction in PAT amplitude with increased pulse rate, (2) reduction in PAT amplitude with a 3% oxygen desaturation, and (3) 4% oxygen desaturation. AHI is calculated from RDI, and the severity is based on the AASM definition (mild: 5-15/h, moderate: 15-30/h, and severe: >30/h). ODI is 4% desaturation events per hour.

Outcomes

As primary outcomes, we compared the AHI, ODI, sleep efficiency, and percentage of REM sleep with and without HFNC therapy. Secondary outcomes included other variables measured by WatchPAT and the patient’s adherence and discomfort to HFNC therapy.

Statistical analysis

Continuous data are presented as mean ± standard deviation or median [interquartile range (IQR)], as appropriate, whereas categorical data are presented as number (%). Variables were compared using a paired t-test and the Wilcoxon signed-rank test. Sample size was not calculated a priori due to a pilot study [12]. Data analyses were conducted using JMP version 13.1.0 (SAS Institute Inc., Cary, NC). All statistical tests were two-tailed, and the chosen type 1 error rate was a p value of <0.05.

## Results

**Figure 1:**
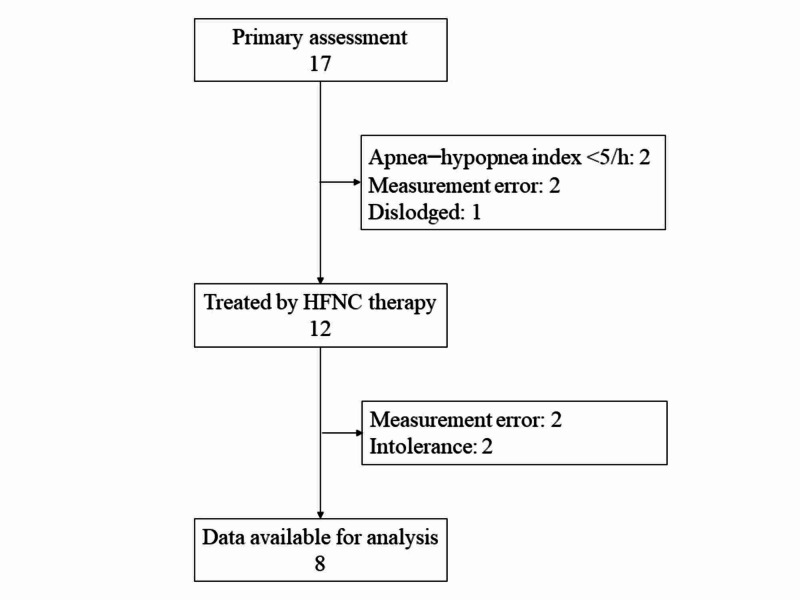
Flowchart of patients included in this study. A total of 17 patients were assessed for sleep apnea syndrome, and 12 patients were included for HFNC therapy. Eight patients remained for final analysis. HFNC: high-flow nasal cannula.

A total of 17 patients were assessed for SDB. Two patients did not meet the inclusion criteria of AHI <5/h. Measurement error was observed in two patients. In one patient, the WatchPAT was dislodged unintentionally during sleep. In total, 12 patients were treated by HFNC at 40 L/min, and one patient was titrated to 30 L/min according to the patient’s acceptance. Among the twelve patients, eight were included for final analysis because two recordings resulted in insufficient data and HFNC therapy was not adhered in two patients due to intolerance (Figure 1).

Table 1 summarizes the characteristics of the study patients.

**Table 1:** Patient characteristics BMI: body mass index, GCS: Glasgow Coma Scale, NIHSS: National Institute of Health Stroke Scale, AHI: apnea-hypopnea index, SD: standard deviation.

Patients	A	B	C	D	E	F	G	H	Mean	SD
Characteristics										
Age, year	79	65	90	57	73	62	50	70	68	12
Gender	M	M	F	M	M	M	M	M		
BMI, kg/m^2^	21.7	28.6	23.6	21.0	21.5	32.2	31.4	20.5	25.0	4.6
GCS	15	15	14	15	14	6	15	15	14	3
NIHSS	10	0	24	8	16	23	1	8	11	8
Sleep disorders										
AHI, evens/h	7.4	7.3	8.0	20.6	17.6	24.7	35.2	49.3	21.3	14.0
Severity	Mild	Mild	Mild	Moderate	Moderate	Moderate	Severe	Severe		

The mean age of the patients was 68 ± 12 years, and seven patients were males. Six patients had ischemic stroke, and one patient had intracerebral hemorrhage (patient E) or subarachnoid hemorrhage (patient B). Only one patient required surgery before the measurement (patient B). The measurement was conducted on day 3 (IQR: 2-4) after the admission to the Stroke Care Unit. Two patients complained of discomfort after the HFNC therapy (patients E and G). During the measurements, the body position was not different between HFNC therapy vs control (spine position: 88% vs 78%, p = 0.39).

**Figure 2:**
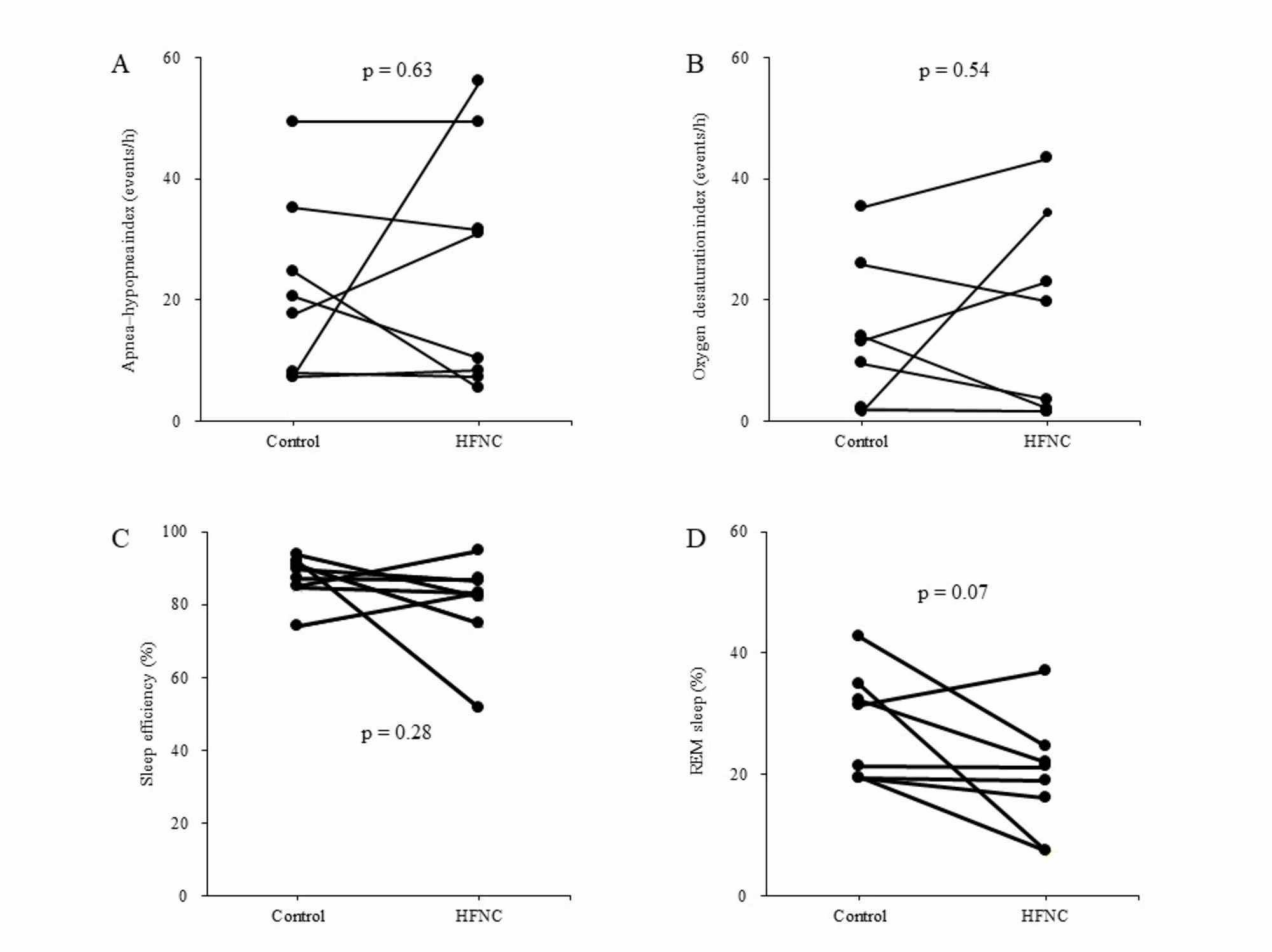
Results of actigraphy before and after HFNC therapy. A, Apnea-hypopnea index. B, Oxygen desaturation index. C, Sleep efficiency. D, REM sleep. No differences were observed between patients with and without HFNC. Data are compared using the Wilcoxon signed-rank test. HFNC: high-flow nasal cannula, REM: rapid eye movement.

There were no differences between patients with and without HFNC therapy in primary outcomes (Figure 2, Table 2).

**Table 2:** Results of actigraphy AHI: apnea-hypopnea index, ODI: oxygen desaturation index, REM: rapid eye movement, HFNC: high-flow nasal cannula, SD: standard deviation.

	AHI (events/h)			ODI (events/h)			Sleep efficiency (%)			REM sleep (%)	
Patients	Control	HFNC		Control	HFNC		Control	HFNC		Control	HFNC
A	7.4	8.3		2.2	1.6		74.0	83.3		19.4	16.1
B	7.3	56.0		1.5	34.5		91.8	51.7		19.6	7.4
C	8.0	7.2		1.8	1.6		93.7	82.2		19.3	19.0
D	20.6	10.3		9.5	3.5		90.9	74.9		32.3	22.0
E	17.6	31.0		13.1	23.0		84.8	83.1		31.3	37.1
F	24.7	5.4		14.0	2.0		89.6	86.5		34.8	7.4
G	35.2	31.6		25.9	19.7		87.0	87.0		42.8	24.6
H	49.3	49.3		35.3	43.3		85.1	94.6		21.4	21.3
Mean	21.3	24.9		12.9	16.2		87.1	80.4		27.6	19.4
SD	15.0	20.1		12.3	16.5		6.2	12.9		8.9	9.6

Regarding other variables, the results for HFNC therapy vs control were as follows: total sleep time 451 ± 75 vs 497 ± 80 min (p = 0.27), percentage of non-REM sleep 79.2% ± 8.3% vs 72.4% ± 8.9% (p = 0.06) , and RDI 25.7 ± 19.6 vs 21.8 ± 14.5/h (p = 0.60). There was no difference in respiratory and circulatory parameters between patients with and without HFNC therapy as follows: SpO_2_ 94.8% ± 1.3% vs 95.1% ± 1.1% (p = 0.53) and heart rate 68 ± 14 vs 68 ± 14/min (p = 0.98).

## Discussion

In the present study, we observed that HFNC therapy did not improve SDB and sleep quality in patients with acute stroke. To our knowledge, this is the first study to investigate the effect of HFNC therapy on SDB and sleep quality in patients with acute stroke. Although HFNC therapy is commonly more acceptable than CPAP ventilation therapy, four (34%) of twelve patients with HFNC therapy complained of discomfort and two patients could not continue to receive HFNC therapy, indicating insufficient adherence to the respiratory therapy.

The end-expiratory pressure generated by HFNC therapy may not be sufficient to prevent the collapse of upper airway. Although we did not measure the end-expiratory pressure, the pharyngeal pressure generated by the HFNC flow of 40 L/min is around 1.5 cm H_2_O [13]. In general, 5-15 cm H_2_O is required to stabilize the symptom of OSA during CPAP ventilation therapy [14,15]. It is understandable that the pressure generated by HFNC therapy was not effective in reducing upper airway collapse. However, McGinley et al. reported that a nasal insufflation of 20 L/min improved upper airway patency [9]. The difference may be attributed to the study population. In our study, we investigated the effect of HFNC therapy in patients with acute stroke. Therefore, central SAS, or at least the complex of central and obstructive, may be included in the subjects. The use of HFNC therapy may negatively affect those patients having central SAS. HFNC therapy washes out nasopharyngeal dead space and consequently reduces carbon dioxide levels. This condition may lead to a poor response from the central nerve and may aggravate the central SAS. Another study also reported a different result of a device between stroke patients and general population. That study used nasal expiratory positive airway pressure, which is a valve inserted into each nostril. The device did not reduce AHI in stroke patients, but it was useful in the general population [16,17].

HFNC therapy did not change the sleep efficiency and the percentage of REM sleep. In stroke patients, sleep quality is often impaired [18]. In a meta-analysis, sleep efficiency was found to be lower in stroke patients than in controls (69%-79% vs 78%-78%, p = 0.006) [19]. Improving sleep quality is essential in stroke patients because poor sleep quality is associated with impaired functional status [20]. However, HFNC therapy was insufficient in improving sleep quality possibly due to unchanged AHI and noise-induced sleep disruption. Although we did not measure the noise level, the noise level generated by HFNC therapy is 50-70 dB at 40 L/min and exceeds the recommended level of <30 dB at night [21,22]. Noise-reducing strategies are required to facilitate sleep promotion during HFNC therapy in the acute care unit [23].

The important finding of this study was not the high acceptance ratio of HFNC therapy. In CPAP ventilation therapy, a low adherence rate of 20%-40% is a problem, and the use of HFNC therapy is expected to have a better compliance rate [8]. However, two patients did not tolerate HFNC therapy, with two more complaining of the discomfort. The discomfort is attributed to the nasal irritation due to the high amount of flow and temperature. In a previous study using 20 L/min of flow through an open nasal cannula, all patients tolerated the flow. The flow of 40 L/min may exceed the patient’s tolerance. In fact, the flow of 30-60 L/min reportedly caused discomfort, especially in the population with less severe acute hypoxemic respiratory failure [24]. In our study, most of the patients had normal lung function, which may be related to the discomfort despite the severity of SDB.

There are several limitations in our study. First, the sample size was small. Second, we did not use polysomnography, which is a gold standard but expensive and difficult to conduct immediately outside of a specialized center, stressful in the head attachment, and difficult to attach on the head after head surgery [25]. In our study, polysomnography was not suited in the early phase of stroke care. In most of the previous studies, WatchPAT correlated with polysomnography (correlation coefficient: 0.87-0.94; p < 0.01), and the sensitivity/specificity values were 100%/75% and 80/100% at an AHI of 5 and 30/h, respectively [26]. Therefore, our data are still reliable. Third, we included stroke patients who may have atherosclerosis in peripheral blood vessels. WatchPAT may underestimate sleep events in these subjects [27].

## Conclusions

We evaluated the effect of HFNC therapy on SDB and sleep quality in patients with acute stroke. HFNC therapy did not change SDB and sleep quality, and nonadherence and discomfort were observed during the therapy. We need a large trial to confirm this result.
